# Antimicrobial Use and Manure Management Among Pig and Poultry Farmers in Malawi

**DOI:** 10.3390/antibiotics14111141

**Published:** 2025-11-11

**Authors:** Amon Abraham, Andrew G. Mtewa, Chimwemwe Chiutula, Richard Lizwe Steven Mvula, Alfred Maluwa, Fasil Ejigu Eregno, John Njalam’mano

**Affiliations:** 1 Department of Energy Resources, Ndata School of Climate and Earth Sciences, Malawi University of Science and Technology, Limbe P.O. Box 5196, Malawi; moh-013-22@must.ac.mw; 2 Department of Applied Studies, Chemistry Section, Malawi Institute of Technology, Malawi University of Science and Technology, Limbe P.O. Box 5196, Malawi; amtewa@must.ac.mw; 3 Department of Earth Sciences, Ndata School of Climate and Earth Sciences, Malawi University of Science and Technology, Limbe P.O. Box 5196, Malawi; poh-004-22@must.ac.mw; 4 Directorate of Research and Outreach, Malawi University of Science and Technology, Limbe P.O. Box 5196, Malawi; aomaluwa@must.ac.mw; 5 Department of Building, Energy and Material Technology, Faculty of Engineering Science and Technology, UiT, The Arctic University of Norway, Postboks 385, 8514 Narvik, Norway; fasil.e.eregno@uit.no; 6 Department of Water Resources Management, Ndata School of Climate and Earth Sciences, Malawi University of Science and Technology, Limbe P.O. Box 5196, Malawi; jnjalammano@must.ac.mw

**Keywords:** antimicrobial resistance, attitudes, knowledge, Malawi, one health, practices, pig farmers, poultry farmers

## Abstract

Background/objectives: Antimicrobial resistance (AMR) is a growing public health concern, and misuse of antibiotics in livestock farming contributes to its emergence. In Blantyre, Malawi, small-scale pig and poultry farming is widespread, but the knowledge, attitudes, and practices (KAP) driving antimicrobial use (AMU) remain poorly understood. This study aimed to assess the KAP regarding AMU and manure management among pig and poultry farmers in Blantyre, Malawi. Methods: This cross-sectional study surveyed 118 randomly selected farmers to assess AMU patterns, sources of antibiotics, adherence to withdrawal periods, disposal practices, and awareness of AMR and regulations. Data was collected using a structured questionnaire and analyzed with descriptive statistics and inferential tests (with statistical significance set at *p* < 0.05). Results: Antibiotic use was reported by 88% of farmers, primarily for therapy (93.3%) and prophylaxis (85.6%), including for viral diseases such as Newcastle disease in poultry and African swine fever in pigs. Oxytetracycline (91.5%), penicillin (50.8%), and trimethoprim-sulfamethoxazole (39.8%) were the most used antibiotics, predominantly sourced from agrovet shops (73.7%). While 61% of farmers knew antibiotic misuse could lead to AMR, significant gaps were observed: 68.6% had no formal training, 55.9% were unaware of regulations, and 42% sold/consumed products before the end of the withdrawal period. Most farmers disposed of expired antibiotics (80.5%) and packaging (92.4%) in household waste. Higher education and prior training were significantly associated with good knowledge. Conclusions: This study reveals significant knowledge–practice gaps and high-risk behaviors, such as misuse for viral diseases and unsafe disposal, that exacerbate AMR risks. Interventions must prioritize targeted farmer education, strengthening of veterinary extension services, and stricter regulation of agrovet shops to promote antimicrobial stewardship and support Malawi’s National Action Plan on AMR.

## 1. Introduction

AMR represents one of the most pressing global public health and development threats of the 21st century. Projections suggest it could cause 10 million annual deaths by 2050 with catastrophic economic damage [1,2]. AMR arises when microorganisms, such as bacteria, develop the ability to withstand the effects of antimicrobials that were previously effective. The emergence and spread of AMR are driven primarily by inappropriate and excessive use of antimicrobials in human medicine and animal production [3,4]. Within agri-food systems, antibiotics are routinely used for therapeutic, prophylactic, and growth-promoting purposes. This creates sustained selective pressure that fuels the development of resistant bacteria [5,6]. These resistant bacteria, along with their genetic determinants, can spread to humans through direct contact with animals, environmental contamination, and the consumption of animal products, completing a critical farm-to-fork AMR transmission pathway [1,7,8]. The challenge of AMR is disproportionately severe in low- and middle-income countries (LMICs). Weak regulatory frameworks, limited access to veterinary services, and high infectious disease burdens perpetuate antimicrobial misuse in these settings [9,10,11]. This is evident in sub-Saharan Africa (SSA), where studies from Ghana, Kenya, and Tanzania consistently report high rates of non-prescription antibiotic access, poor farmer knowledge, and inappropriate usage practices [12,13,14].

Malawi, located in southeastern Africa, exemplifies these challenges. The country’s small-scale pig and poultry sectors have expanded rapidly to meet rising demand for animal-source protein [15,16]. These production systems are characterized by limited biosecurity, high disease incidence, and a heavy reliance on antimicrobials to prevent economic losses. Studies in Malawi have confirmed high antibiotic usage in broiler production, with oxytetracycline, erythromycin, and enrofloxacin being frequently used, largely driven by disease outbreaks and poor biosecurity [17,18]. Recognizing this threat, Malawi developed a National Action Plan (NAP) on AMR in 2017, emphasizing the need for rational antimicrobial use across human and animal health sectors [19]. However, its implementation faces challenges, including inconsistent funding, human resource shortages, and a weak regulatory environment [20]. A critical barrier to effective intervention is a lack of detailed, on-the-ground data on the drivers of antimicrobial use at the farm level.

Recent research in Malawi has begun to illuminate the complex drivers and consequences of antimicrobial misuse [17,18,21,22,23]. A study by Mankhomwa et al. [17] reveals a precarious reliance on antibiotics, where they are deemed vital for economic survival in small-scale intensive farming systems characterized by limited veterinary oversight. This dependency is facilitated by the easy over-the-counter availability of WHO Critically Important Antimicrobials (CIAs), including colistin [17,21]. The environmental impact of these practices has been confirmed by the detection of multidrug-resistant *E. coli* and *K. pneumoniae* in manure, soil, and vegetables from local farms in Blantyre City, Malawi, creating a direct farm-to-fork transmission risk [23]. This problem is exacerbated by a fundamental disconnect within the animal healthcare system. Although a recent study by Kainga et al. [22] found that Malawian veterinarians and para-veterinarians possess good knowledge and attitudes towards antimicrobial stewardship, but their expertise often does not reach the primary decision-makers, the farmers. Instead, farmers frequently rely on informal advice and source antibiotics from agrovet shops, where a separate study revealed dispensers had overall poor KAP regarding antimicrobial use and resistance [21].

Therefore, while previous research has documented usage patterns [17,18], environmental contamination [23], and the knowledge of veterinarians [22] and agrovet staff [21], a crucial evidence gap persists. A comprehensive, quantitative baseline assessment of the Knowledge, Attitudes, and Practices (KAP) of the primary decision-makers, the farmers themselves, is currently lacking. Specifically, there is no integrated study that directly links farmer behaviors regarding antibiotic use and manure management to the documented AMR risks in the farm environment. To bridge this gap, we conducted a focused cross-sectional KAP survey among pig and poultry farmers in Blantyre, Malawi. The novelty of this work lies in its integrated approach, providing the first quantitative baseline that connects specific farmer KAP with manure management practices, thereby offering a holistic view of the AMR transmission pathway at the farm level.

Therefore, this study aimed to conduct a detailed cross-sectional KAP survey among pig and poultry farmers in Blantyre, Malawi. Specifically, we sought to: (1) quantify the patterns and types of antimicrobials and other health products used; (2) identify the primary sources of antibiotics and assess adherence to administration guidelines; (3) evaluate farmers’ knowledge and attitudes regarding AMU and AMR; and (4) document critical practices related to withdrawal periods and the disposal of antibiotics and waste. By establishing this integrated behavioral baseline, our findings will provide essential evidence to inform the development of context-specific, farmer-focused educational campaigns and policies, ultimately supporting the national and global goals of mitigating AMR through a One Health approach.

## 2. Results

### 2.1. Sociodemographic Characteristics of the Study Participants

A total of 118 farmers participated in the study. The majority were male (55%, 65/118) and aged between 36–49 years (42%, 50/118). Educational attainment was moderately high, with 40% (47/118) having completed secondary school and another 40% (47/118) having attained tertiary education. In terms of farming operations, 46% (54/118) were engaged in poultry farming, 43% (51/118) in pig farming, and 11% (13/118) managed both types of livestock. The average farming experience was 3.8 years (3.1 ± SD), with most farmers (81%, 95/118) having 1–5 years of experience. Table 1 presents the demographic characteristics of the study participants.

### 2.2. Antibiotics Prophylactic Use and Misapplication for Viral Diseases

**Figure 1:**
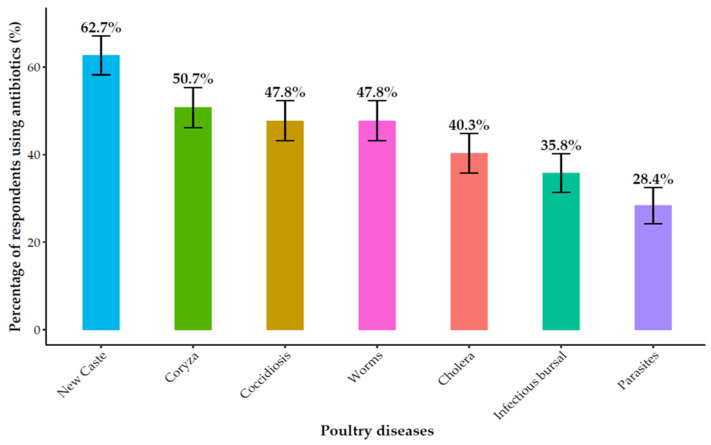
Antibiotic use for common poultry diseases.

**Figure 2:**
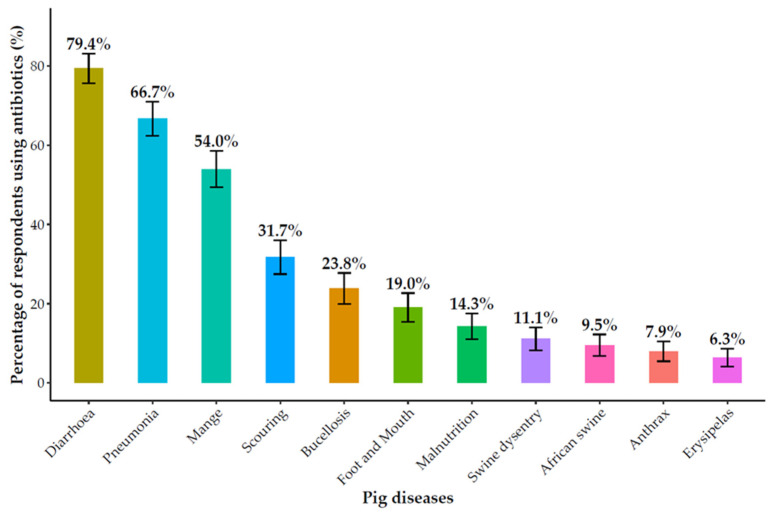
Antibiotics used for common pig diseases.

Antibiotics constituted the primary method of animal health management, used by 88.1% (104/118) of respondents (Figure S1). Their application was primarily for therapeutic (93.3%, 97/104) and prophylactic (85.6%, 89/104) purposes, with a smaller subset (16.9%, 18/104) reporting use for growth promotion. Of particular concern was the widespread misuse of antibiotics for viral diseases, which can contribute to antimicrobial resistance (AMR). In poultry, antibiotics were most frequently used to treat Newcastle disease (62.7%, 42/67), infectious coryza (50.7%, 34/67), and coccidiosis (47.8%, 32/67) (Figure 1). In pigs, they were used predominantly for diarrhea (79.4%, 50/63), pneumonia (66.7%, 42/63), and mange (54.0%, 34/63), as well as for African swine fever (9.5%, 6/63) (Figure 2).

Beyond antibiotics, vaccines (68.6%, 81/118) and herbal remedies (37.3%, 44/118) were commonly used livestock health interventions. The use of these products showed significant demographic variations. Female farmers were significantly more likely to use herbal remedies than males (47.2%, 25/53 vs. 29.2%, 19/65, *p* < 0.05). Conversely, vaccine adoption was significantly higher among poultry farmers (94.4%, 51/54) compared to pig farmers (37.3%, 19/51) (*p* < 0.05). Furthermore, respondents with tertiary education were significantly more likely to use both vaccines (*p* < 0.05) and herbal remedies (*p* < 0.05) compared to those with lower educational attainment (Table 2). This demographic variation highlights potential targets for AMR awareness and training interventions. The associations between demographic characteristics and the use of herbal remedies and vaccines are presented in Table 2.

### 2.3. Antibiotic Sourcing, Administration, and Adherence Practices

**Figure 3:**
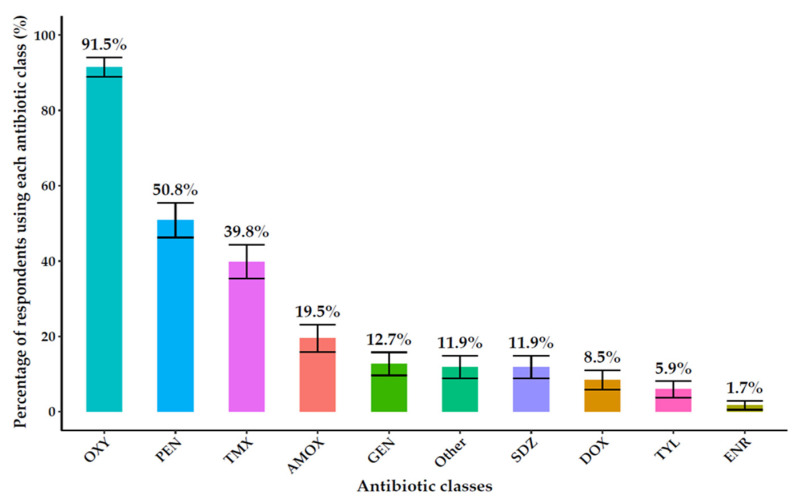
Types of antibiotics used on farms include oxytetracycline (OXY), penicillin (PEN), trimethoprim-sulfamethoxazole (TMX), amoxicillin (AMOX), gentamycin (GEN) sulfadiazine (SDZ), doxycycline (DOX), tylosin (TYL), enrofloxacin (ENR).

The most reported antibiotic classes were oxytetracycline (91.5%, 108/118), penicillin (50.8%, 60/118), and trimethoprim-sulfamethoxazole (39.8%, 47/118). Farmers primarily sourced antibiotics from agrovet shops (73.7%, 87/118) and veterinarians (61%, 72/118) (Figure 3). The prevalence of different antibiotic classes reported by farmers is shown in Figure 3.

Consultation with veterinary professionals was reported by 86% (101/118) of farmers who always or sometimes consulted a veterinarian before administration. However, a significant gender disparity was observed: male farmers were significantly more likely to seek veterinary consultation than female farmers (95%, 62/65 vs. 81% 43/53, *p* < 0.05).

The responsibility for administering antibiotics was distributed among veterinarians/animal health workers (63%, 74/118), farm owners (57%, 68/118), and farm workers (13%, 16/118), and other farmers (7%,8/118). Overall, self-reported adherence to administration guidelines was generally high: 85.6% (101/118) of farmers reported following manufacturer’s instructions or veterinary prescriptions, and 89.8% (106/118) reported always checking antibiotic expiry dates. However, critical deviations were noted. A concerning 30.5% (36/118) of farmers reported they would increase the dosage or frequency of antibiotics if animals did not improve, and 20.3% (24/118) would stop administration immediately upon observing animal improvement.

Adherence was strongly influenced by education and farm type. Nearly all respondents with tertiary education followed drug prescriptions (98%, 46/47) and checked expiry dates (100%, 47/47), rates significantly higher than those with lower education levels (*p* < 0.001). Similarly, poultry farmers demonstrated significantly better adherence to prescription (96%, 52/54 vs. 75%, 38/51, *p* < 0.05) and expiry date checking (98%, 53/54 vs. 82%, 42/51, *p* < 0.05) compared to pig farmers. The decision to discontinue treatment early was significantly associated with a lack of formal education (71%, 5/7 vs. 15–19% for educated groups, *p* < 0.05) (Tables S1–S3).

### 2.4. Knowledge and Attitudes Regarding Antibiotic Use and AMR

Assessment of knowledge revealed that 61% (72/118) of farmers were aware that antibiotic misuse could lead to AMR, while 36.4% were unaware, and 2.5% (3/118) believed it did not contribute to AMR. The majority (89%, 105/118) recognized the importance of consulting a veterinarian and following drug prescriptions (86.4%, 102/118). Most farmers lacked formal training on antibiotic use (68.6%, 81/118), and more than half (55.9%, 66/118) were unaware of regulations governing antibiotic use. The overall knowledge, attitude, and practice indicators among the surveyed farmers are summarized in Table 3.

Awareness of AMR as a problem was significantly higher among farmers with tertiary education (74%) compared to those with primary (41%, 7/17), secondary (47%, 22/47), or no formal education (43%, 3/7) (*p* < 0.05). A significant gender disparity was also found in regulatory awareness, with male farmers being more aware than female farmers (54%, 35/65 vs. 32%, 17/53, *p* < 0.05) (Tables S4 and S5). Awareness of AMR and regulations is thus strongly linked to education and gender, indicating priority groups for training and policy enforcement. Training participation was significantly higher among those with tertiary education (38%, 18/47), poultry farmers (44%, 24/54), and older farmers (>50 years, 45%, 17/38) (*p* < 0.05) (Table 4). The influence of demographic factors on awareness of AMR as a problem, regulatory knowledge, and training participation is detailed in Tables S3–S5.

### 2.5. Practices and Knowledge on Withdrawal Periods

About 65% (77/118) of respondents were aware of antibiotic withdrawal periods, while 35% (41/118) were unfamiliar. Additionally, 64% (75/118) recognized the importance of adhering to withdrawal periods, whereas 36% (43/118) did not (Table 5). Veterinary advice was the most common method for determining withdrawal periods (58%, 68/118), followed by guidance from agro-vet shop staff (42%, 49/118) and manufacturer’s instructions (33%, 39/118). However, 17% (20/118) were uncertain about how to determine these periods. In practice, 42% (50/118) of respondents admitted to selling or consuming animal products before completing the withdrawal period, 38% (45/118) adhered to the recommendations, and 19% (23/118) were unsure about compliance (Table 5). Non-adherence exposes consumers to antibiotic residues, increasing AMR risk and food safety concerns. Table 5 summarizes the respondents’ knowledge and practices concerning the antibiotic withdrawal period.

Awareness of withdrawal periods was significantly higher among farmers with tertiary education (79%, 37/47) compared to those with no formal education (14%, 1/7) (*p* < 0.05) (Table S6).

### 2.6. Disposal Practices for Antibiotics and Packaging

The majority of farmers disposed of expired antibiotics (80.5%, 95/118) and empty antibiotic packaging (92.4% 109/118) in regular household waste. While a subset of farmers employed safer disposal methods like returning to suppliers (21.2%, 25/118) or incineration (9.3%, 11/118 for antibiotics, 8.5%, 10/118 for packaging), a small but concerning proportion admitted to disposal in open fields or water bodies (3.4%, 4/118 for antibiotics, 2.5%, 3/118 for packaging) (Table 6). Improper disposal of antibiotics can lead to environmental contamination, further propagating antimicrobial resistance. The methods used for the disposal of expired antibiotics and their packaging are detailed in Table 6.

### 2.7. Manure Management Practices

The manure management practices among respondents showed considerable variation, with 51.69% (61/118) storing manure for later use and 50% (59/118) composting it. Other methods included spreading manure directly on fields (13.56%, 16/118) or disposing of it in landfills (11.9%, 14/118). Only 3.4% (4/118) reported using alternative methods (Table S7). Manure management is a key factor in AMR risk, as antibiotic residues and resistant bacteria may persist in stored manure. The majority of respondents (78.8%,93/118) stored manure for more than two weeks before its use or disposal, while 11% (13/118) stored it for 1–2 weeks, 10.2% (12/118) did not store manure, and 2.5% (3/118) stored it for less than one week (Table 7). When asked about the potential impact of manure from treated animals on soil or crop health, 39.8% (47/118) were unsure, 30.5% (36/118) believed there was no effect, and 29.7% (35/118) felt that it could have an impact. Table 7 summarizes the duration for which respondents store manure before its use or disposal, providing insights into common manure management practices.

### 2.8. Antibiotics Available in Veterinary Shops

A survey of five veterinary shops in Blantyre revealed a wide range of antibiotics commonly used in livestock and poultry production. The antibiotics identified include Penicillin-based drugs (e.g., Procaine Penicillin, Amoxicillin), Oxytetracycline (available in injectable, powder, and spray forms), Tylosin, Doxycycline, Sulpha-based antibiotics (e.g., Sulfadimidine, Sulphaquinoxaline), Colistin, Gentamicin, Enrofloxacin (a fluoroquinolone), and Trimethoprim-Sulphamethoxazole combinations. Additionally, Chlortetracycline and Neomycin were also found, often formulated with vitamins for enhanced efficacy. These antibiotics are used to treat a variety of infections, including respiratory, gastrointestinal, urinary tract, and soft tissue infections, as well as coccidiosis and mycoplasmosis, in livestock such as cattle, pigs, sheep, goats, and poultry. Veterinary shops serve as major points of antibiotic distribution, highlighting opportunities for stewardship interventions to reduce misuse.

## 3. Discussion

This study provides critical information, of the KAP that underpin antibiotic use and antimicrobial resistance (AMR) among pig and poultry farmers in Blantyre, Malawi. The findings reveal a high-risk ecosystem characterized by pervasive antibiotic dependence, significant knowledge–practice gaps, and risky habits that directly contribute to the environmental dissemination of AMR. This study links farmers’ behaviors with environmental contamination patterns, complementing our companion study that detected multidrug-resistant bacteria in manure, soil, and vegetables from the same farming systems [23]. Together, these findings strengthen evidence for a farm-to-fork AMR transmission pathway.

### 3.1. Structural and Economic Drivers of Pervasive Antibiotic Misuse

The very high prevalence of antibiotic use (88%) establishes it as the cornerstone of animal health management in this setting. This finding is consistent with patterns across sub-Saharan Africa, where antibiotics are a default intervention in smallholder systems facing high disease burdens [24,25]. Similar trends have been reported in Malawi [18], Ghana (97%) [13], Kenya (80%) [14], Zambia (83%) [26], Burkina Faso (32%) [27] and Tanzania (97.1%) [28]. The high rate of prophylactic use (85.6%) is particularly alarming, as it creates constant selective pressure for resistance. This practice should not be seen simply as a knowledge gap. It is an economically rational yet high-risk response to production pressures. Farmers operate under conditions of high stock density, poor biosecurity, and significant financial vulnerability. In such contexts, the loss of even a few animals can cause substantial economic setbacks. This makes pre-emptive antibiotic use a perceived necessity for risk mitigation [17,29], effectively functioning as a low-cost insurance policy, a behavior observed across multiple African production systems [13,14,26,28].

This economic logic is reinforced by structural constraints. Access to veterinary diagnostics and professional services is limited. The widespread misapplication of antibiotics for viral diseases such as Newcastle disease in poultry and African swine fever in pigs reflects this gap. Without affordable veterinary guidance or diagnostic tools [9,17], farmers and agrovet staff effectively assume the role of primary decision-makers in diagnosis and treatment. This leads to antibiotics being used as a blanket response to undifferentiated illness a pattern documented in other low-resource settings [14,30]. This approach not only wastes scarce resources but also accelerates resistance, creating a double burden of treatment failure and increased risk of resistant infections in both animals and humans.

### 3.2. The Agrovet Nexus: Filling a Knowledge and Regulation Void

The predominant reliance on agrovet shops as the primary antibiotic source for 73.7% of farmers indicates their critical, yet problematic, role in the animal healthcare ecosystem. Given the limited availability and affordability of formal veterinary services, farmers increasingly depend on these retail outlets as their first point of contact for both medical advice and treatments. Although agrovet staff are often valued for their practical experience with livestock diseases and treatments, many lack formal veterinary training, which frequently leads to inconsistent or inappropriate recommendations. This pattern aligns with broader trends observed across LMICs, where agrovet shops have evolved into primary animal health hubs due to persistent veterinary shortages [9,31,32].

The dependence on informal antibiotic sources extends beyond Malawi’s borders, reflecting a regional challenge. In western Kenya, for instance, more than half of agrovet staff lacked formal qualifications while 40% of antibiotics were dispensed without prescriptions [32]. Similarly, studies from Tanzania [24], Uganda [33], Burkina Faso [27], and Rwanda [34] have documented comparable patterns where socioeconomic and geographic barriers, coupled with weak legal enforcement and limited veterinary extension services, perpetuate informal antimicrobial access. Particularly concerning is the documented availability of colistin, a last-resort antibiotic in unregulated agrovet sales in Malawi [17], highlighting the significant public health risks associated with inadequate oversight.

Economic considerations further exacerbate this dynamic, as smallholder farmers facing recurrent disease outbreaks and limited diagnostic capabilities often view inexpensive, readily accessible antibiotics as the most pragmatic solution. This pragmatic dependence perpetuates use of WHO Critically Important Antimicrobials (CIAs) such as oxytetracycline, penicillin, and trimethoprim-sulfamethoxazole, which emerged as the most frequently reported antibiotics in our study. Consequently, antibiotic selection reflects commercial availability and dispenser preference rather than evidence-based decision-making, thereby creating substantial long-term risks for both animal productivity and human health [35].

Our analysis further indicates that women farmers may experience disproportionate impacts from these systemic gaps due to intersecting socioeconomic factors. Cultural norms, financial constraints, and limited mobility often restrict women’s access to formal veterinary services, rendering them more dependent on informal advice from agrovet shops or peers [36,37]. Such structural disadvantages explain the observed gender disparities in regulatory awareness and veterinary consultation. Hence, AMR mitigation must incorporate gender-responsive strategies that empower women with equitable access to training and veterinary information.

To address these challenges, strengthening the agrovet nexus through multifaceted interventions becomes imperative. Priority actions should include building agrovet staff capacity through structured antimicrobial stewardship training, enforcing prescription-only antibiotic access, and better integrating veterinary extension services. Additionally, leveraging digital health platforms and targeted community awareness programs could reduce reliance on informal advice, as demonstrated by successful mobile-based livestock programs in Malawi [18]. Addressing these structural issues is therefore essential to transform agrovets from unregulated dispensers into partners in antimicrobial stewardship.

### 3.3. Bridging the Knowledge–Practice Gap: Behavioral Drivers and Systemic Failures

This study reveals a persistent disconnect between knowledge and practice in antibiotic use among livestock farmers. While 61% of respondents recognized that misuse contributes to antimicrobial resistance (AMR), 69% had received no formal training, leaving them without the practical competence required for correct implementation. This void in actionable knowledge, widely reported across sub-Saharan Africa [27,34,38], drives dependence on informal information networks and perpetuates cycles of misuse and misinformation.

These knowledge gaps manifest in high-risk behaviors that are best understood as rational adaptations to economic pressure rather than irrational neglect. The tendency to escalate antibiotic doses (30.5%) or prematurely discontinue treatment (20.3%) represents coping strategies aimed at minimizing financial loss and achieving rapid recovery [39]. Likewise, the disregard for withdrawal periods reported by 42% of farmers poses a direct public health threat through consumer exposure to antibiotic residues. This pattern, driven by economic necessity and limited health awareness, mirrors systemic issues documented in Tanzania and Burkina Faso [27,28] and is corroborated by studies detecting antibiotic residues in food products [40,41].

At a structural level, these behaviors are reinforced by the absence of safe disposal infrastructure. The disposal of over 80% of expired antibiotics and 90% of packaging in household waste creates environmental contamination hotspots [42,43]. When combined with inadequate manure management, such as the application of poorly composted manure, these practices complete a pathway for resistant bacteria and genes to enter the wider ecosystem [44,45]. This pathway directly links on-farm behaviors to the environmental contamination confirmed in our companion study [23]. While education and literacy remain foundational to effective antimicrobial stewardship [4,46], awareness alone is insufficient. Sustainable change requires integrated interventions that address both behavioral drivers—through practical, hands-on training and farmer engagement—and systemic failures, by improving veterinary extension services, ensuring affordable waste disposal options, and strengthening regulatory enforcement. Only through this dual approach can behavioral change translate into measurable reductions in AMR risk within smallholder livestock systems.

### 3.4. Promising Alternatives and Structural Barriers: Vaccines, Herbs, and Gender

Amid these risks, the study identifies crucial opportunities for intervention. The significant use of vaccines (69%) and herbal remedies (37%) indicates an existing farmer appetite for preventive and alternative health measures. The higher vaccine uptake among poultry farmers and those with higher education suggests that awareness and industry integration can successfully reduce antibiotic dependence, a cornerstone of global AMR strategies [47].

This study reveals critical intervention opportunities alongside persistent structural challenges. The substantial adoption of vaccines (69%) and herbal remedies (37%) demonstrates a strong foundation for promoting preventive and alternative health measures among farmers. The particularly high vaccine uptake among poultry farmers indicates that sector-specific approaches and industry integration can effectively reduce antibiotic dependence, aligning with core global AMR mitigation strategies [47].

The use of herbs like *Moringa oleifera*, *Aloe vera*, *Hibiscus sabdariffa*, *Solanum incanum*, *Zingiber officinale*, *Allium sativum*, and charcoal reflects valuable indigenous knowledge and a desire for “natural” alternatives, a trend documented in Malawi and Zimbabwe [17,48]. This is also common in Ghana, where 40% of domestic poultry farmers administer herbs [13]. However, this is a double-edged sword. While some botanicals possess confirmed bioactive properties [49], the efficacy and safety of many local remedies are unvalidated. Over-reliance on them can lead to treatment failure and may ultimately drive farmers to use higher doses of antibiotics when alternatives fail, underscoring an urgent need for research to validate and standardize effective herbal treatments.

Economic pressures and structural barriers perpetuate these challenges. The imperative to reduce costs and maintain productivity strongly influences farmers’ reliance on self-treatment and informal remedies, exacerbated by limited access to veterinary services. These factors intersect with gender disparity: male farmers were significantly more likely to consult veterinarians and be aware of regulations, which suggests that women face systemic barriers to accessing knowledge and formal services [36,37]. This disparity is not unique to Malawi; in Nepal, male gender was a positive predictor of good knowledge and practices [50], and in Cameroon, gender was negatively associated with the practice of antimicrobial use [51]. In Rwanda, sex was also significantly correlated with both knowledge and attitudes, reinforcing the need for gender-sensitive interventions to ensure equitable access to training and veterinary services [34]. This disparity must be addressed through gender-sensitive extension programs to ensure inclusive and effective AMR mitigation.

A key finding that offers a clear path forward is the contrast between the low farmer training (69% had none) and the high levels of knowledge and good attitudes found among Malawian veterinarians and para-veterinarians [22]. This identifies the country’s veterinary professionals as a major, yet underutilized, asset in the fight against AMR. As Kainga et al. [22] conclude, these professionals “can be entrusted to comply with responsible antimicrobial prescriptions and use,” making them the ideal cornerstone for any future educational intervention and antimicrobial stewardship program targeting farmers.

### 3.5. Study Limitations

This study has several limitations. Its cross-sectional design captures knowledge, attitudes, and practices at a single point, limiting causal inference and observation of behavioral changes over time. Data were self-reported and subject to recall and social desirability biases, particularly regarding antibiotic use, withdrawal adherence, and disposal practices, despite training and confidentiality assurances. The focus on pig and poultry farmers in selected urban and peri-urban areas of Blantyre may limit generalizability to other livestock systems or rural regions with different socioeconomic and cultural contexts. Although a companion environmental study confirmed multidrug-resistant bacteria in related systems, behavioral data were not linked directly to microbiological or residue testing, restricting analysis of AMR transmission pathways. Information on herbal and alternative treatments was self-reported and unverified by pharmacological analyses. Gender and socioeconomic representation were uneven, with women and lower-income farmers underrepresented. Despite these limitations, the study provides a critical behavioral baseline and identifies priorities for future research, including longitudinal designs, expanded geographic scope, and integration of microbiological evidence within One Health frameworks.

## 4. Materials and Methods

### 4.1. Study Design and Area

**Figure 4:**
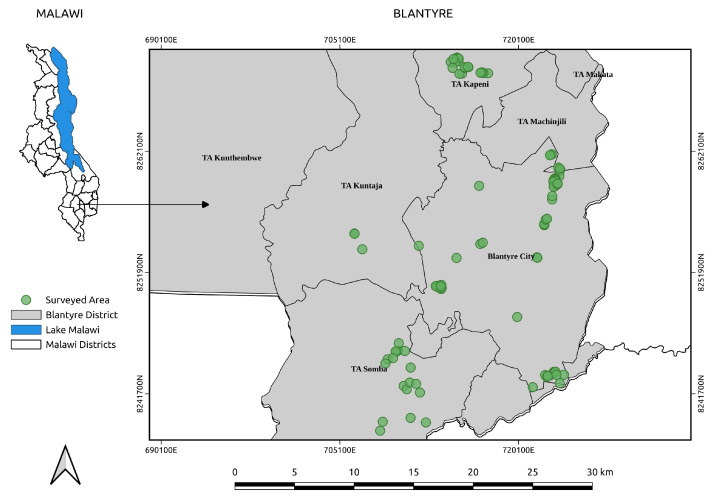
Map of Blantyre City showing surveyed farms.

A cross-sectional study was conducted from August to December 2024 in urban and peri-urban areas of Blantyre City, Malawi. The study area lies in the Shire Highlands (15°47′05″ S; 35°00′30″ E) and experiences a subtropical climate with an annual rainfall of 1122 mm and temperatures ranging from 13 °C to 21 °C, conditions conducive to intensive livestock farming [52]. To capture a representative snapshot of urban and peri-urban livestock practices, the study focused on three of Blantyre’s agricultural Extension Planning Areas (EPAs): Ntonda, Chipande, and Kunthembwe. Within these EPAs, data collection was carried out in specific areas, including Manase, Mpemba, Baluti, Kampala, and Bangwe (Ntonda EPA); Machinjiri, Mbayani, Chilimba, and Ndirande (Chipande EPA); and Chileka (Kunthembwe EPA) (Figure 4). This multi-site approach was designed to capture the diversity of urban and peri-urban agricultural practices in Blantyre.

### 4.2. Sample Size and Sampling

The minimum required sample size was calculated as 97 farmers using the Epitools online calculator for estimating a proportion. The calculation was based on an expected antibiotic use prevalence of 20%, a desired margin of error of 8% (selected to balance statistical precision with practical feasibility in this resource-limited setting), and a 95% confidence level (z-score of 1.96), parameters derived from prior studies in similar contexts [31,53]. The calculated sample size was increased to 118 to account for potential non-response and to enhance the statistical power of the study. Participant selection followed a rigorous two-stage process to minimize selection bias. First, a preliminary list of potential farmers was obtained from the Blantyre District Agricultural Office. Second, this list was thoroughly reviewed and corrected by Assistant Veterinary Officers (AVOs) based on their extensive field knowledge. The AVOs verified the status of listed farmers and added active farmers who were not formally registered, ensuring the final sampling frame accurately reflected the mixed reality of both formal and informal livestock operations in the study area. From this validated and comprehensive list, 118 participants were randomly selected for inclusion in the study.

### 4.3. Data Collection

Data were collected through face-to-face interviews using a structured questionnaire (Section S10) adapted from previously validated tools used in similar contexts in Zambia and Ghana [26,31]. Key sections on knowledge, attitudes, and practices regarding antibiotic use and resistance were adapted from these instruments. To ensure cultural and linguistic appropriateness the questionnaire was translated into Chichewa, the local language, and the translated version was back-translated into English by an independent translator to ensure conceptual equivalence. It was then pre-tested with 10 farmers (not included in the main study) to assess clarity, appropriateness, and cultural relevance. The internal consistency of the Likert-scale knowledge and attitude items was assessed using Cronbach’s alpha, which was 0.78, indicating acceptable reliability. The final instrument was digitally administered using the KoBoToolbox platform on smartphones to ensure data accuracy and integrity. The questionnaire comprised six sections covering sociodemographic characteristics, antibiotic usage patterns, knowledge and attitudes toward antibiotic use, practices regarding antibiotic withdrawal periods, antibiotic and packaging disposal practices, and manure handling and management practices. Eight trained research assistants conducted the interviews and ensured consistency in data collection procedures.

### 4.4. Data Analysis

All statistical analyses were performed using R software (version 4.5.0). A comprehensive data cleaning process was undertaken, and all coding was documented in reproducible scripts to ensure transparency and replicability. Descriptive statistics, including frequencies, proportions, means, and standard deviations, were used to summarize the data. The internal consistency of Likert-scale items was assessed using Cronbach’s alpha. For inferential analysis, associations between sociodemographic factors and knowledge, attitude, and practice outcomes were analyzed using Chi-square tests or Fisher’s exact tests for categorical variables, while one-way ANOVA with post hoc Tukey HSD tests was used to compare mean knowledge, attitude, and practice scores across different farmer groups, including farming type (pig, poultry, or mixed), education level, and gender. Given the exploratory nature of this study aimed at identifying potential associations for future research, no adjustments for multiple comparisons were made, as the goal was to identify potential associations for future research rather than to test specific a priori hypotheses. Therefore, *p*-values should be interpreted with caution. A *p*-value of < 0.05 was considered statistically significant for all analyses.

## 5. Conclusions

This study provides the first comprehensive quantitative baseline of knowledge, attitudes, and practices regarding antimicrobial use and manure management among pig and poultry farmers in Blantyre, Malawi, directly linking specific high-risk behaviors to AMR environmental contamination pathways. The findings reveal that despite moderate awareness, a critical knowledge–practice gap persists, driven by structural issues, including limited training, gender disparities, and easy access to antibiotics through informal channels. To effectively mitigate AMR risks, we recommend three prioritized, actionable interventions: (1) implementing targeted, gender-sensitive education programs for farmers and capacity-building for agrovet staff; (2) strengthening enforcement of prescription-only antibiotic access and veterinary extension services; and (3) developing practical systems for safe pharmaceutical waste disposal and proper manure management. Future research should focus on evaluating the impact of these interventions, validating the efficacy and safety of commonly used herbal alternatives, and exploring the economic drivers that influence farmer decision-making. These focused actions and research priorities are essential to support Malawi’s National Action Plan on AMR and safeguard both animal and public health.

## Figures and Tables

**Table 1:** Demographic characteristics of the study respondents (*N* = 118).

Variable	Category	*n* (%)
Gender	Male	65 (55)
	Female	53 (45)
Age	21–35	30 (25)
	36–49	50 (42)
	>50	38 (32)
Education level	No formal education	7 (6)
	Primary school	17 (14)
	Secondary school	47 (40)
	Tertiary education	47 (40)
Farm type	Pig Farm	51 (43)
	Poultry farm	54 (46)
	Both	13 (11)
Years spent farming	1–5	95 (81)
	6–10	17 (14)
	11–15	5 (4.2)
	16+	1 (0.8)
$\mathrm{Years}\;\mathrm{spent}\;\mathrm{farming}\;(\bar{x}$ ± SD)	years farming	117 (3.80 ± 3.14)

**Table 2:** Association between demographic characteristics and the use of herbal remedies and vaccines for livestock health management.

	Herbals			Vaccine		
Variable	No	Yes	*p*-Value	No	Yes	*p*-Value
Education			*p* < 0.05			*p* < 0.05
No formal education	2 (2.7%)	5 (11%)		4 (11%)	3 (3.7%)	
Primary school	13 (18%)	4 (9.1%)		10 (27%)	7 (8.6%)	
Secondary school	25 (34%)	22 (50%)		8 (22%)	39 (48%)	
Tertiary education	34 (46%)	13 (30%)		15 (41%)	32 (40%)	
Gender			*p* < 0.05			
Male	46 (62%)	19 (43%)				
Female	28 (38%)	25 (57%)				
Farm type						*p* < 0.05
Pig Farm				32 (86%)	19 (23%)	
Poultry farm				3 (8.1%)	51 (63%)	
Both				2 (5.4%)	11 (14%)	

**Table 3:** Knowledge, attitude, and practice indicators related to antibiotic use among farmers.

Variable	Yes (%)	No (%)	I Don’t Know (%)	*p*-Value
AMR is a problem	94	6	-	*p* < 0.05
I check expiry before the drug administration	90	10	-	*p* < 0.05
I consult a veterinarian	89	1	10	*p* < 0.05
I follow drug prescription	86	14	-	*p* < 0.05
I stop drug administration when I see improvement in my animal’s health	20	80	-	*p* < 0.05
I Increased the dosage or frequency of antibiotic treatment when animals did not improve.	31	69	-	*p* < 0.05
I know that misuse of drugs leads to AMR	61	3	36	*p* < 0.05
Aware of regulations on antibiotic use	44	56	-	*p* < 0.05
I was trained on antibiotic usage	31	69	-	*p* < 0.05
I observe drug withdraw periods	65	35	-	*p* < 0.05

**Table 4:** Training on antibiotic use by demographic factors (gender, age, education, and farm type).

Characteristic	Category	N	Training Usage	p-Value
Age group	21–35	30 ^1^	5 (17)	*p* < 0.05
	36–49	50 ^1^	15 (30)	
	>50	38 ^1^	17 (45)	
Education level	No Formal Education	7 ^1^	0 (0)	*p* < 0.05
	Primary School	17 ^1^	1 (5.9)	
	Secondary School	47 ^1^	18 (38)	
	Tertiary Education	47 ^1^	18 (38)	
Farm type	Pig Farm	51 ^1^	9 (18)	*p* < 0.05
	Poultry Farm	54 ^1^	24 (44)	
	Both	13 ^1^	4 (31%)	

^1^ *n* (%).

**Table 5:** Respondents’ knowledge and practices regarding antibiotic withdrawal periods.

Statement	Response	*n* (%)
Aware of the withdrawal period	Yes	77 (65)
	No	41 (35)
Understand the importance of withdrawal periods.	Yes	75 (64)
	No	43 (36)
Determine of withdrawal period	Veterinary advice	68 (58)
	Agrovet shop staff advice	49 (42)
	Manufacturer’s instructions	39 (33)
	Advice from other farmers	17 (14)
	Personal experience	10 (8)
	I don’t know	20 (17)
Sold or consumed products before the withdrawal period	Yes	50 (42)
	No	45 (38)
	I don’t know	23 (19)

**Table 6:** Disposal practices for expired antibiotics and empty antibiotic packaging among respondents (*N* = 118).

Disposal Method	Expired Antibiotics *n* (%)	Empty Packaging *n* (%)
Disposal in regular waste	81 (95)	109 (92)
Return to supplier	21 (25)	-
Incineration	9 (11)	10 (8)
Disposal in irregular waste (open field or water body)	3.4 (4)	3 (3)
Other (toilet)	2 (2)	-

**Table 7:** Manure storage duration among respondents (*N* = 118).

Manure Storage Duration	*n* (%)
More than two weeks	98 (78.8%)
1–2 weeks	13 (11%)
I don’t store manure	12 (10.2%)
Less than one week	3 (2.5%)

Note: Extended storage duration can affect the survival of antibiotic residues and resistant bacteria, influencing AMR risks when manure is applied to land.

## Data Availability

The original contributions presented in this study are included in the article.

## Supplementary Materials

The following supporting information can be downloaded at: https://www.mdpi.com/article/10.3390/antibiotics14111141/s1. Figure S1. Types of health products (antibiotics, vaccines, and herbal remedies) used by pig and poultry farmers in animal health management. Table S1. Adherence to antibiotic prescription guidelines by education level and farm type. Table S2. Adherence to expiry date checking guidelines by education level and farm type. Table S3. Early discontinuation of antibiotic treatment by education level. Table S4. Awareness of antibiotic resistance as a problem by education level. Table S5. Awareness of antibiotic use regulations by gender. Table S6. Awareness and Adherence to Antibiotic Withdrawal Periods by Education Level. Table S7. Manure management practices among respondents (N = 118). Section S9: Participants informed consent. Section S10: Questionnaire: Antibiotic Usage Practices among Pig and Poultry Farmers.
