# Supplementary material for: Antimicrobial Use and Manure Management Among Pig and Poultry Farmers in Malawi
Source: Antibiotics (Basel). 2025 Nov 11;14(11):1141. doi: 10.3390/antibiotics14111141 (PMC12649420; doi:10.3390/antibiotics14111141)
Supplement: Supplementary file 1 [file antibiotics-14-01141-s001.zip › antibiotics-3892009-supplementary.pdf]

Supplementary Materials

Section S1

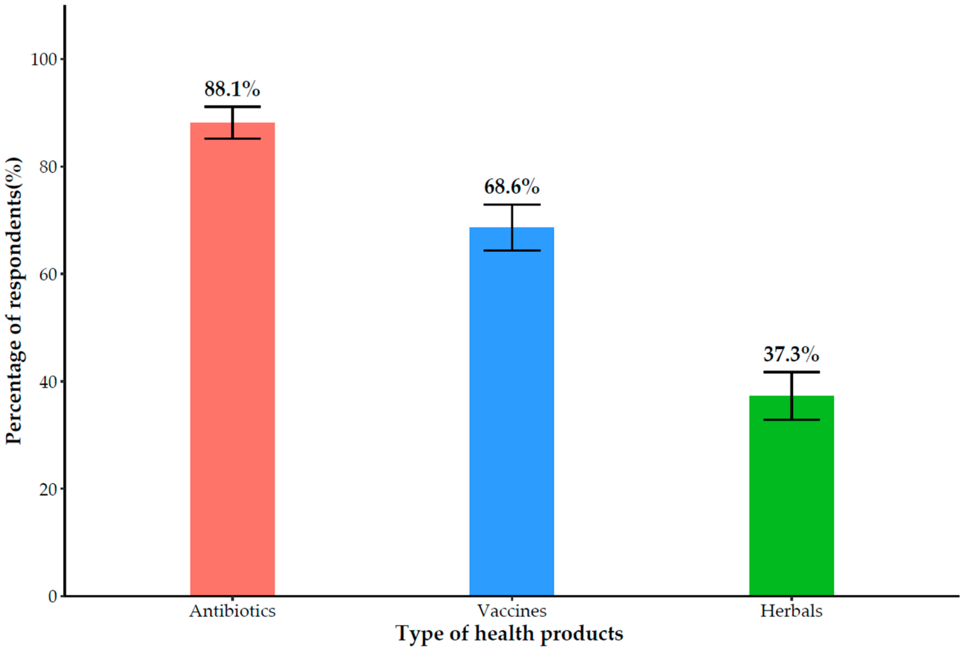

Figure S1. Types of health products (antibiotics, vaccines, and herbal remedies) used by pig and poultry farmers in animal health management.

Section S2

Table S1. Adherence to antibiotic prescription guidelines by education level and farm type.

Characteristic	Category	N	Follow prescription	p-value
Education Level	No formal education	7[1]	3 (43%)	$p<0.05$
	Primary school	17[1]	10 (59%)	
	Secondary school	47[1]	42 (89%)	
	Tertiary education	47[1]	46 (98%)	
Farm Type	Pig Farm	51[1]	38 (75%)	$p<0.05$
	Poultry farm	54[1]	52 (96%)	
	Both,	13[1]	11 (85%)	

Section S3

Table S2. Adherence to expiry date checking guidelines by education level and farm type.

Characteristic	Category	N	Check expiry	p-value
Education Level	No formal education	71[1]	3 (43%)	p<0.05
	Primary school	17[1]	11 (65%)	
	Secondary school	47[1]	46 (98%)	
	Tertiary education	47[1]	46 (100%)	
Farm Type	Pig Farm	51[1]	41 (82%)	p<0.05
	Poultry Farm	54[1]	53 (98%)	
	Both	13[1]	12 (92%)	

[1]n (%)

Section S4

Table S3. Early discontinuation of antibiotic treatment by education level.

Characteristic	Tertiary education, N = 47[1]	Primary school, N = 17[1]	Secondary school, N = 47[1]	No formal education, N = 7[1]	p-value[2]
Stop antibiotic administration once there is improvement	9 (19)	3 (18)	7 (15)	5 (71)	p<0.05

[1]n (%)

Section S5

Table S4. Awareness of antibiotic resistance as a problem by education level.

Characteristic	No formal education, N = 7[1]	Primary school, N = 17[1]	Secondary school, N = 47[1]	Tertiary education, N = 47[1]	p-value[2]
AMR is a problem					$p<0.05$
Yes	3 (43)	7 (41)	22 (47)	35 (74)	
I don't know	4 (57)	9 (53)	23 (49)	11 (23)	
No	0 (0)	1 (5.9)	2 (4.3)	1 (2.1)	

[1]n (%)

Section S6

Table S5. Awareness of antibiotic use regulations by gender.

Characteristic	Male, N = 65[1]	Female, N = 53[1]	p-value
Aware of antibiotic use regulations	35 (54)	17 (32)	$p<0.05$

[1]n (%)

Section S7

Table S6. Awareness and Adherence to Antibiotic Withdrawal Periods by Education Level.

Characteristic	Tertiary education, N = 47[1]	Primary school, N = 17[1]	Secondary school, N = 47[1]	No formal education, N = 7[1]	p-value[2]
Aware of the antibiotic withdrawal period	37 (79)	11 (65)	28 (60)	1 (14)	$p<0.05$

[1]n (%)

Section S8

Table S7. Manure management practices among respondents (N=118)

Manure Management Practice	n (%)
Storing for later use	61 (51.7)
Composting	59 (50)
Spreading directly on fields	16 (13.6)
Disposal in landfills	14 (11.9)
Other methods	4 (3.4)

Section S9

Title of Research Project: Knowledge, Attitudes, and Practices on Antibiotic Use and Manure Management among Pig and Poultry Farmers in Blantyre, Malawi

Details of principal investigator (pi):

Title, first name, surname:

Mr. Amon Abraham

MUSTREC reference number: P.03/2021/121

Full postal and email address: Malawi University of Science and Technology, P.O. Box 5196, Limbe, Malawi;

Amonabraham7@gmail.com/ moh-015-22@must.ac.mw

PI Contact number: +265 881209311/+265 994545487

We would like to invite you to take part in a research project. Please take some time to read the information presented here, which will explain the details of this project. Please ask the study staff or doctor any questions about any part of this project that you do not fully understand. It is very important that you are completely satisfied that you clearly understand what this research entails and how you could be involved. Also, your participation is entirely voluntary, and you are free to decline to participate. In other words, you may choose to take part, or you may choose not to take part. Nothing bad will come of it if you say no: it will not affect you negatively in any way whatsoever. Refusal to participate will involve no penalty or loss of benefits or reduction in the level of care to which you are otherwise entitled to. You are also free to withdraw from the study at any point, even if you do agree to take part initially.

This study was approved by the Malawi University of Science and Technology Research Ethics Committee (MUSTREC). The study will be conducted according to the ethical guidelines and

principles of the International Declaration of Helsinki, CIOMS Guidelines, Belmont Report, Guidelines for Good Clinical Practice, and Ethical Guidelines for Research issued by the National Commission for Science and Technology.

What is this research study all about?

This study aims to understand the knowledge, attitudes, and practices of pig and poultry farmers in Blantyre regarding antibiotic use and how they manage animal manure. We want to learn about the choices farmers make to help improve animal health and farming practices.

Why are we doing this study?

Antibiotics are important for keeping animals healthy, but their misuse can lead to drug resistance, making infections harder to treat. How manure is managed can also affect the environment and health. This study will help us understand current practices so we can develop better training and guidelines to support farmers in Malawi.

Why do we invite you to participate?

You are invited because your experience as a pig or poultry farmer in Blantyre is invaluable. Your insights will help us truly understand the situation on the ground and create useful recommendations for other farmers and policymakers.

What will your responsibilities be?

If you agree to participate, a researcher will conduct a confidential interview with you. The interview will ask questions about:

- Your farm and background.
- How you use antibiotics for your animals (e.g., why, how often, where you get them).
- Your knowledge and opinions about antibiotics and drug resistance.
- How you handle and dispose of animal manure.
- The interview will take about 20-30 minutes of your time.

Will you benefit from taking part in this research?

There are no direct financial benefits. However, your participation will help improve knowledge and develop better support programs for farmers in Malawi, which could benefit you and your community in the future.

Are there risks involved in your taking part in this research?

There are no physical risks. The main risk is a potential loss of privacy. We will minimize this by keeping your information completely confidential and anonymous. Your name will not be recorded on the questionnaire or used in any report.

If you do not agree to take part, what alternatives do you have?

If you do not agree to take part in this research study, you have the option to continue with your usual agricultural practices without participating in the study. This means you will not provide any information regarding the antibiotic use and manure management on your farm. You are free to make this decision without any negative consequences or penalties.

Who will have access to your records?

The information you provide is strictly confidential. Your name will not be used anywhere. The data will be stored securely and only the research team will have access to it. The results will be published in a way that no individual can be identified.

What will happen in the unlikely event of some form of injury occurring as a direct result of your taking part in this research study?

The risk of injury from participating in this study is extremely low. Since the involvement only requires granting permission for data collection, there are minimal procedures involved. However, if an injury occurs during collection, we will provide first aid, help you get medical care, and cover any associated costs. Please ask any questions about risks or injuries before deciding.

Will you be paid to take part in this study and are there any costs involved?

Your participation in this study is entirely voluntary and there will be no compensation involved. We appreciate your time and contribution to this important research.

Is there anything else that you should know or do?

➢ You can phone Mr. Amon Abraham at 0881209311 if you have any further queries about the study or encounter any problems during your participation in the study.

➢ You can contact the MUSTREC Administrator at 01 478 000 ext 8253 or mustrec@must.ac.mw if you have any concerns or complaints that have not been adequately addressed by the researcher.

➢ You will receive a copy of this information and a consent form for you to keep safe.

Declaration by participant

By signing below, I …………………………………..…………. agree to take part in a research study entitled "Impact of Antibiotic Residues in Manure on the Presence of Antibiotic-Resistant Bacteria in Agricultural Soils and Vegetables "

I declare that:

- I have read this information and consent form, or it was read to me, and it is written in a language in which I am fluent and with which I am comfortable.

- I have had a chance to ask questions and I am satisfied that all my questions have been answered.

- I understand that taking part in this study is voluntary, and I have not been pressurized to take part.

- I may choose to leave the study at any time and nothing bad will come of it – I will not be penalised or prejudiced in any way.

- I may be asked to leave the study before it has finished, if the study doctor or researcher feels it is in my best interests, or if I do not follow the study plan that we have agreed on.

Signed at (place) ....….......…………….. on (date) …………...……….. 2021.

Signature of participant

Declaration by investigator/researcher

I (name) ……………………………………………..……… declare that:

- I explained the information in this document to ………………………………..

- I encouraged him/her to ask questions and took adequate time to answer them.

- I am satisfied that he/she adequately understands all aspects of the research, as discussed above.

Signed at (place) ....….......…………….. on (date) …………...……….. 2021.

Signature of investigator/researcher

For an illiterate participant

A literate impartial witness must sign (if possible, this person should be selected by the participant and should have no connection to the research team). Illiterate participants should include their thumbprint as well.

I have witnessed the accurate reading of the consent form to the potential participant, and the individual has had the opportunity to ask questions. I confirm that the individual has given consent freely.

Print name of witness: ....

Signature of witness: ....

Thumb print of participant

Date: ______________________

Day/month/year

Section S10

Questionnaire: Antibiotic Usage Practices among Pig and Poultry Farmers

Section A: General Information

1. Gender:

☐ Male

☐ Female

2. Age (years):

☐ <20

☐ 21 - 35

☐ 36 - 50

☐ >50

3. Education Level:

☐ No formal education

☐ Primary school

☐ Secondary school

☐ Tertiary education

4. Farm Type:

☐ Pig farm

☐ Poultry farm

☐ Both

5. Farm Size:

☐ Small-scale (1-50 animals/birds)

☐ Medium-scale (51-200 animals/birds)

☐ Large-scale (200+ animals/birds)

Section B: Antibiotic Usage

6. Are antibiotics used on your farm?

☐ Yes

☐ No

7. For what purposes are antibiotics used on your farm? (Select all that apply)

☐ To treat diseases

☐ To prevent diseases

☐ For growth promotion

☐ Other (please specify): ___________

8. Which diseases are antibiotics used for?

☐ Bacterial diseases

☐ Viral diseases

☐ any disease

☐ Don't know

9. How often are antibiotics administered to the animals/birds on your farm?

☐ Regularly

☐ Occasionally

☐ Only when animals/birds are sick

10. Do you consult with an animal health professional for antibiotic use?

☐ Always

☐ Sometimes

☐ Never

11. Where do you usually purchase antibiotics?

☐ Veterinarian

☐ Agrovet shop

☐ Human pharmacy

☐ Other (please specify): ___________

12. Are the manufacturer's instructions or veterinary prescriptions followed when using antibiotics?

☐ Yes

☐ No

☐ Experience from previous knowledge

13. Do you check the expiry date of the antibiotics when administering them on your farm?

☐ Yes

☐ No

14. I increase the dose of antibiotics and frequency of administration if animals do not show any signs of recovery.

☐ Yes

☐ No

15. If animals feel better after the first day of treatment, I stop giving the antibiotics.

☐ Yes

☐ No

16. I consider the recommendations of other farmers about antibiotic use.

☐ Yes

☐ No

Section C: Knowledge and Attitudes

17. Do you think misuse or overuse of antibiotics can lead to antibiotic resistance?

☐ Yes

☐ No

☐ I don't know

18. Have you received any training or information on the proper use of antibiotics?

☐ Yes

☐ No

19. Do you think it is important to consult a veterinarian before using antibiotics?

☐ Yes

☐ No

☐ I don't know

20. Do you believe that antibiotic resistance is a problem that should be prevented?

☐ Yes

☐ No

☐ I don't know

21. Are you aware of any regulations or guidelines on antibiotic usage in livestock farming?

☐ Yes

☐ No

Section D: Antibiotic Withdrawal Periods

22. Are you aware of the term "withdrawal period" concerning antibiotic use?

☐ Yes

☐ No

23. Do you understand the importance of adhering to withdrawal periods after administering antibiotics to your animals?

☐ Yes

☐ No

24. How do you determine the appropriate withdrawal period for the antibiotics you use? (Select all that apply)

☐ Manufacturer's instructions

☐ Veterinary advice

☐ Personal experience

☐ I do not know

25. Have you ever sold or consumed products (meat, eggs, milk) from animals before the withdrawal period was completed?

☐ Yes

☐ No

☐ I don't know

Section E: Antibiotic and Packaging Disposal

26. How do you dispose of expired antibiotics?

☐ Return to supplier

☐ Incineration

☐ Disposal in regular waste

☐ Other (please specify): ___________

27. How do you dispose of empty antibiotic packaging?

☐ Recycling

☐ Incineration

☐ Disposal in regular waste

☐ Other (please specify): ___________

28. Are there any measures in place to safely dispose of antibiotic waste?

☐ Yes

☐ No

If yes, please specify: _______________

Section F: Manure Handling

29. How do you typically manage manure from your livestock? (Select all that apply)

☐ Composting

☐ Spreading directly on fields

☐ Storing for later use

☐ Disposing of it in a landfill

☐ Other (please specify): ___________

30. How long do you usually store manure before using or disposing of it?

☐ Less than one week

☐ 1-2 weeks

☐ More than two weeks

☐ I do not store manure

31. Do you believe that using manure from treated animals can affect the health of your crops or soil?

☐ Yes

☐ No

☐ I don't know
